# Repeated mild traumatic brain injuries induce persistent changes in plasma protein and magnetic resonance imaging biomarkers in the rat

**DOI:** 10.1038/s41598-019-51267-w

**Published:** 2019-10-10

**Authors:** David K. Wright, Rhys D. Brady, Alaa Kamnaksh, Jack Trezise, Mujun Sun, Stuart J. McDonald, Richelle Mychasiuk, Scott C. Kolbe, Meng Law, Leigh A. Johnston, Terence J. O’Brien, Denes V. Agoston, Sandy R. Shultz

**Affiliations:** 1 Department of Neuroscience, Central Clinical School, Monash University, Melbourne, VIC 3004 Australia; 2 The Florey Institute of Neuroscience and Mental Health, The University of Melbourne, Melbourne, VIC 3052 Australia; 3 Department of Medicine, The Royal Melbourne Hospital, The University of Melbourne, Melbourne, VIC 3052 Australia; 4 Department of Anatomy, Physiology, and Genetics, Uniformed Services University, Bethesda, MD 20814 USA; 5 Department of Biomedical Engineering, The University of Melbourne, Melbourne, VIC 3052 Australia

**Keywords:** Diagnostic markers, Brain injuries

## Abstract

A single mild traumatic brain injury (mTBI) typically causes only transient symptoms, but repeated mTBI (RmTBI) is associated with cumulative and chronic neurological abnormalities. Clinical management of mTBI is challenging due to the heterogeneous, subjective and transient nature of symptoms, and thus would be aided by objective biomarkers. Promising biomarkers including advanced magnetic resonance imaging (MRI) and plasma levels of select proteins were examined here in a rat model of RmTBI. Rats received either two mild fluid percussion or sham injuries administered five days apart. Rats underwent MRI and behavioral testing 1, 3, 5, 7, and 30 days after the second injury and blood samples were collected on days 1, 7, and 30. Structural and diffusion-weighted MRI revealed that RmTBI rats had abnormalities in the cortex and corpus callosum. Proteomic analysis of plasma found that RmTBI rats had abnormalities in markers indicating axonal and vascular injury, metabolic and mitochondrial dysfunction, and glial reactivity. These changes occurred in the presence of ongoing cognitive and sensorimotor deficits in the RmTBI rats. Our findings demonstrate that RmTBI can result in chronic neurological abnormalities, provide insight into potential contributing pathophysiological mechanisms, and supports the use of MRI and plasma protein measures as RmTBI biomarkers.

## Introduction

Mild traumatic brain injuries (mTBI), such as concussions, are induced by biomechanical forces acting on the brain, and are a common and significant health problem worldwide^1^. A single mTBI can result in a range of neurological impairments that are typically transient^2^. On the other hand, repeated mTBIs (RmTBI), which are particularly common in contact sports and military settings, have been associated with significant and lasting neurological abnormalities, and have been indicated as a risk factor for developing various neurodegenerative diseases^3–5^.

The heterogeneous and subjective nature of mTBI and RmTBI symptomatologies present serious challenges in the clinical management of these injuries^6,7^. As such, the identification of objective biomarkers for these injuries has become a research priority in recent years^8–10^. Previous preclinical and clinical findings have found that advanced magnetic resonance imaging (MRI) and blood-based protein measures are promising and clinically applicable mTBI biomarkers^8–13^. For instance, diffusion-weighted MRI (DWI) methods, such as diffusion tensor imaging (DTI) and tractography, detect the diffusion of water molecules within the brain and are sensitive to pathophysiological changes, such as axonal injury, that can be induced by mTBI^12,14–16^. Blood-based protein biomarkers are also capable of detecting a number of pathobiological changes that may occur following mTBI, including metabolic, neuronal, axonal, glial, inflammatory, and vascular changes^11,12,17–20^. Of particular relevance to this study, we have previously found that a single mild fluid percussion injury (mFPI) in rats results in abnormalities detectable by both DWI and plasma protein measures, and that these changes persisted longer than the cognitive abnormalities that recovered by day five post-mFPI^12^.

Although there is growing evidence that MRI and blood-based protein measures can detect changes after a single mTBI, how RmTBI affects these measures and how they relate to the functional consequences of RmTBI remains largely unknown relative to the growing understanding of a single mTBI. This is in part due to the difficulties in rigorously studying the effects of RmTBI and characterizing biomarkers purely in the clinical setting^21,22^. In particular, investigating the effects and evolution of clinical RmTBI present additional challenges and complexities (e.g., inter-injury time) than studies of a single mTBI. Furthermore, as RmTBI is quite common in athletes and soldiers, it is important to understand how RmTBI might influence biomarkers differently than an initial mTBI. Therefore, in this study we used the repeated mFPI (RmFPI) rat model to determine changes detected by MRI, select plasma protein biomarkers, and neurobehavioral measures at different recovery times after RmTBI.

## Methods

### Subjects

Forty-eight male Long-Evans rats were purchased from Monash animal research services (Melbourne, Australia). All rats were 8–12 weeks of age and weighed 250–300 g at time of injury. Following surgery, rats were housed individually under a 12 h:12 h light/dark cycle with ad libitum access to water and food for the duration of the study. All experimental procedures were approved by The University of Melbourne and The Florey Institute of Neuroscience and Mental Health animal ethics committees, and complied with the guidelines of the Australian Code of Practice for the Care and Use of Animals for Scientific Purposes. It is important to note that data collection for this study was done in conjunction with another study that examined behavior, blood, and MRI biomarkers after a single mFPI [12]. This was done in an effort to reduce animal usage, and therefore only a single group of sham-injured rats was used. Consequently, there is some overlap between the sham data presented in this paper and our previous paper [12].

### Repeated mild lateral fluid percussion injury (RmFPI)

Our laboratory, and others, have previously demonstrated that a single mFPI induces temporary behavioral and pathophysiological abnormalities^23–25^, while RmFPI that are separated by 5 days induce cumulative and chronic pathological and behavioral changes^26–29^. These abnormalities resemble the neurological consequences that mTBI and RmTBI patients may experience, and provides the rationale for the use of the RmFPI model in this study.

All of the procedures used were based on standard protocols as previously described^12,26–29^. Briefly, under isoflurane induced anesthesia a craniotomy (5 mm diameter) was performed (anterior/posterior: −3.0 mm; medial/lateral: 4.0 mm, relative to Bregma) to expose the intact dura and a hollow injury cap was attached to the skull over the craniotomy. Rats were then attached to the fluid percussion device via the head cap. At the first sign of hind-limb withdrawal to a toe-pinch, rats received a fluid percussion pulse of 1–1.5 atm before being disconnected from the device. Sham-injury rats underwent the same procedures except the fluid injury pulse was not administered. Topical antibiotic ointment was then applied, and a sterile plug was inserted into the injury cap to seal the craniotomy. Five days following the initial injury, rats were anaesthetized for their second mFPI or sham injury. The plug was removed from the cap, the cap was filled with saline and the rat was connected to the FPI-device. At first response of hind limb withdrawal sham rats were removed from the device and RmTBI rats received an FPI 1–1.5 atm.

Acute injury measures were monitored after each injury, as shown in Table 1. These included apnea duration (i.e., the time from injury to the return of spontaneous breathing), loss of consciousness (i.e., time to hind limb withdrawal in response to a pinch test), and self-righting (i.e., the time from injury to assuming an upright position)^12,30–32^.

**Table 1:** Acute injury measures after sham and mFPI injuries.

	Sham 1	Sham 2	mFPI 1	mFPI 2
Apnea	0	0	0	0.29 ± 0.29
Hind-limb	0	0	1.47 ± 0.82*	3.82 ± 1.32*
Self-righting	90.69 ± 8.75	82.00 ± 4.00	147.24 ± 9.48*	169.12 ± 6.57*

Repeated measures ANOVA found a significant main effect for injury on the measures of hind-limb and self-righting reflex times, with the mFPI rats showing longer hind-limb and self-righting times. *mFPI longer than sham, *p < *0.05. Numbers indicate mean time (seconds) ± SEM.

### Experimental design

Following their last assigned mFPI or sham injury, rats were allocated to separate behavior (sham = 8; mFPI = 8) or MRI (sham = 8; mFPI = 10) cohorts. The behavior cohort underwent behavioral testing on days 1 (D1), 3 (D3), 5 (D5), and 7 (D7) after the second injury before being euthanized on D7 for blood collection. The MRI cohort underwent MRI at D1, D3, D5, D7 and D30 after the second injury before being euthanized on D30 for blood collection. Note that the MRI cohort also underwent behavioral testing on the day prior to their D30 MRI. This study design allowed us to maximize the amount of data collected on corresponding post-injury days, and also avoided the confounding factor of anesthesia (required for MRI) on behavioral outcomes. An additional 14 rats (sham = 6; mFPI = 8) were euthanized at D1 for blood collection/plasma protein analysis. Thus, plasma samples were available for D1, D7, and D30 post-injury.

### Behavioral testing

A researcher blinded to experimental conditions conducted all behavioral testing. Cognitive function was assessed using the water maze^12^. The water maze apparatus consisted of a circular tank (163 cm in diameter) filled with water (29 °C), and a hidden acrylic escape platform (10 cm diameter) submerged 2 cm below the water surface in one of the quadrants of the pool. Unique visual cues were positioned at the north, south, east and west quadrants. Both the position of the escape platform, and the visual cues, were randomly assigned each day to maintain novelty throughout the course of the experiment. Rats completed four trials per testing day, with the entry position randomized between north, south, east and west for each trial. If the rat failed to find the platform in the allotted minute, then they were placed on the platform for 15 s. Ethovision behavioral tracking software analyzed video from an overhead camera positioned at the center of the tank. Search time (i.e., a measure of cognition), as well as swim speed (i.e., a measure of locomotion), were quantified for each trial.

Sensorimotor function was assessed using a 1 m long, 2 cm wide elevated wooden beam as previously described^12^. Baseline training was performed prior to surgery and consisted of five successful trials on a 4 cm wide beam, followed by five successful trials on a 2 cm wide beam. On each day of testing, rats completed ten trials on the 2 cm wide beam, and the time taken to traverse the beam and the numbers of slips and falls were recorded. The maximum time allowed per trial was 60 s, which was also assigned to rats that fell.

Locomotor activity was assessed using a circular open field (diameter 100 cm, 40 cm high wall) as previously described^12^. Ethovision behavioral tracking software (Noldus, Netherlands) analyzed video from an overhead camera positioned at the center of the field. A single 5 minute trial was performed on each day of testing with the rat positioned in the center of the field. The total distance travelled was analyzed as a measure of locomotion.

Anxiety-like behavior was assessed using an elevated plus maze, as described previously^12^. The maze consists of both open and closed arms, 110 cm long and 12 cm wide, perpendicular to each other, and dividing each in half to form a cross. Fifty cm high walls enclosed the closed arms while the open arms contained no walls. A single 5 minute trial was performed on each day of testing with the rat initially positioned at the center of the cross facing an open arm. Ethovision behavioral tracking software (Noldus, Netherlands) analyzed video from an overhead camera positioned at the center of the cross. The percentage of time spent within the open arms (i.e., measure of anxiety-like behavior), as well as the total distance travelled (i.e., measure of locomotion), were calculated.

### Magnetic resonance imaging (MRI)

The MRI cohort underwent imaging on D1, D3, D5, D7 and D30 as described previously^12^. Anesthesia was induced using 4% Isoflurane in a 1:1 mixture of medical grade air and oxygen. Following loss of consciousness, rats were positioned on a purpose-built animal holder with ear and bite bars to immobilize the head, and a nose cone to deliver Isoflurane to maintain anesthesia. An anatomically shaped 4-chanel surface receive coil was positioned directly over the rat’s head and affixed to the animal holder which was then inserted into an 86 mm volume transmit coil for imaging with a Bruker 4.7/30 MRI.

T_2_^*^-weighted structural images were acquired using a 2D multi-gradient echo sequence with the following imaging parameters: repetition time (TR) = 4400 ms; number of echoes = 8; echo times (TE): 7.5, 15, 22.5, …, 60 ms; field of view (FOV) = 28.8 × 28.8 mm^2^; matrix size = 160 × 160; number of slices = 64; and slice thickness = 180 μm giving an isotropic spatial resolution of 180 × 180 × 180 μm^3^.

DWI was performed using a 2D echo planar-based sequence with the following imaging parameters: TR/TE = 6,000/35 ms; FOV = 25.6 × 25.6 mm^2^; matrix size = 128 × 128; number of slices = 24; slice thickness = 600 μm; diffusion duration (δ) = 3.5 ms; diffusion gradient separation (Δ) = 14 ms; and b-value = 1200 s/mm^2^. Diffusion weighting was performed in 81 directions and 8 non-diffusion-weighted (b0) images were also acquired.

### MRI analysis

T_2_^*^-weighted echo images were averaged across echoes and templates generated at each time point for each cohort using Advanced Normalization Tools (ANTs, http://stnava.github.io/ANTs/). The resulting templates were then combined, again with ANTs, to create a study template. To assess for RmTBI-induced atrophy/hypertrophy, each rat’s mean echo image was then registered to the study template and a tensor-based morphometry (TBM) analysis was performed as previously described^33^.

Diffusion tensor images were calculated for each rat and registered to a study template using the Diffusion Tensor Imaging ToolKit (DTI-TK, http://dti-tk.sourceforge.net/) as described previously^12^. Regions of interest (ROI) were traced on the study template to identify the ipsilateral and contralateral corpus callosum. For each ROI, the mean FA, radial diffusivity (RD), axial diffusivity (AD) and trace (TR) were calculated using FSLstats, part of the FMRIB software library (https://fsl.fmrib.ox.ac.uk/fsl/fslwiki/FSL).

Tractography was performed as described previously using the MRtrix software package (http://www.mrtrix.org/)^12^. Briefly, fibre orientation distribution images were calculated from normalized diffusion images using constrained spherical deconvolution with a group average single-fibre response function. Whole brain tractograms were generated from 2 million streamlines and registered to the study DTI template to normalize both the length and spatial location of the streamlines. Three track-weighted images were generated from the normalized tractograms: average pathlength map (APM), reflecting the average length of all streamlines passing through each voxel^34^; mean curvature map, representing the average curvature of all tracks passing through each voxel^12^; and the track density image (TDI), which maps the total number of streamlines passing through each voxel^35^. As with the DTI measures, the mean of each track-weighted image was calculated for the ROIs.

### Plasma proteomics

Following the completion of experiments on D1, D7, or D30, rats were deeply anaesthetized by an intraperitoneal injection of 0.5 mL sodium pentobarbital (Lethabarb, Virbac, Australia). The chest cavity was then opened and blood was collected into a BD Vacutainer K2 EDTA (K2E) Plus Blood collection tube via cardiac puncture. The tubes were then centrifuged at 6,000 g for 15 min at room temperature, and plasma was pipetted into 0.5 mL aliquots, flash-frozen in liquid nitrogen, and stored at −80 °C until use.

Plasma levels of ceruloplasmin (i.e., a marker for metabolic abnormalities)^19,20,36^, tau protein (i.e., a marker for axonal injury)^19,20,37^, vascular endothelial growth factor (VEGF; i.e., a marker for vascular changes)^19,20,38^, 4-hydroxynonenal Michael adducts (4-HNE; i.e., a marker of oxidative stress)^19,20,39^, NF-H (i.e., a marker of axonal injury)^40^, neuron-specific enolase (NSE; i.e., a marker for neuronal injury)^19,20,41^, glial fibrillary acidic protein (GFAP; i.e., a marker of glial injury/reactivity)^19,20,42^, and S100 calcium binding protein β subunit (S100β; i.e., a marker of astroglia injury)^19,20,42,43^, were assayed using reverse phase protein microarray (RPPM).

Sample preparation, printing, scanning, and data analysis for RPPM were performed as described previously^12^. Frozen plasma samples were thawed on ice and diluted 1:10 with Dilution Buffer (3 parts Lysis Buffer [TPER, 10% Glycerol, 1x HALT] and 1 part 4x SDS Sample Buffer [35% Glycerol, 0.8% SDS, 10x TBS, 10x TCEP, 1x HALT, 0.0035% NaN3]). Samples were then transferred to a JANUS Varispan Integrator and Expanded Platform Workstation (PerkinElmer, Waltham, MA) to perform 1:1 serial dilutions using Dilution Buffer in 384 well microarray plates (product no. X7022; Molecular Devices, Sunnyvale, CA). An equal amount of 2x PBS Buffer (80% Glycerol, 2x PBS) was added to the volume of liquid in each well. The microarray plates were subsequently transferred into an Aushon 2470 Arrayer (Aushon Biosystems, Billerica, MA) where plasma samples were printed onto ONCYTE AVID nitrocellulose film slides (product no. 305177; Grace Bio-Labs, Bend, OR). The Aushon 2470 Arrayer was set up with 16 pins and programmed for 2 depositions per spot. The spot diameter was set to 250 nm with spacing between dots at 500 nm on the x-axis and 375 nm on the y-axis. Wash time was set to 2 s without delays. After overnight desiccation at 4 °C, the slides were blocked with a solution of 1x TBS, 5% non-fat dry milk, and 0.1% Tween-20.

Slides were then incubated with the primary antibody solutions and a cover slip (product no. 25 × 60I-M-5439-001-LS; mSeries LifterSlip; Thermo Fisher Scientific, Waltham, MA) overnight at 4 °C. The primary antibodies were diluted in antibody incubation buffer (0.1% BSA, EDTA-free Halt Protease and Phosphatase Inhibitor Cocktail [Thermo Fisher Scientific], 1x TBS, and 0.5% Tween-20) and used in the following dilutions: ceruloplasmin (1:20; Santa Cruz Biotechnology, sc-21240), NF-H (1:100; Sigma–Aldrich, N4142), tau (1:100; Cell Signaling Technology, 4019), VEGF (1:50; Abcam, ab53465), 4-HNE (1:1000; EMD Millipore, 393207), NSE (1:50; Abcam, ab53025), GFAP (1:20; Abcam, ab48050), and S100β (1:20; Abcam, ab41548). The following day, slides were washed three times with TBST and then incubated with the appropriate secondary antibody solutions for 1 h at room temperature. The secondary antibodies Alexa Fluor 790 goat anti-rabbit (catalog no. A-11369), 680 rabbit anti-goat (catalog no. A-21088), and 680 goat anti-mouse (catalog no. A-21058) (Invitrogen, Eugene, OR) were used at a 1:20,000 dilution. After three thorough washes with TBST followed by a single wash with 1x TBS, the slides were air dried and subsequently scanned in an Innopsys InnoScan 710 IR microarray scanner (Innopsys, Carbonne, France).

Scanner fluorescence data were imported into a Microsoft Excel-based bioinformatics program. After correcting for local background noise, points indiscernible from background were excluded (SNR <2, Net Fluorescence <10). Net intensity vs. dilution was plotted on a log-log scale. A five-parameter logistic (5PL) master curve was fitted to the slide. Using this master curve, outliers were excluded using the False Discovery Rate method (Q = 0.01) and the master curve is further refined. Each local block of samples is fit individually, using master curve values as the initial conditions for the local curve fit. The asymptotic maximum and minimum, Hill-Slope, and asymmetry constant were shared between samples. The slope of the linear portion of the logistic curve was calculated and the line extrapolated back to zero (i.e., the y-intercept), assessing the amount of protein expressed. Data (y-intercept values) are presented as the mean ± S.E.M.

### Statistical analysis

With the exception of the TBM analysis (see “MRI Analysis” section) and the acute injury measures presented in Table 1, all behavior, MRI, and plasma protein measures were analyzed with two-way analysis of variance (ANOVA), with injury and recovery time as the between-subjects factors, using SPSS Statistics 22 software (IBM, New York, NY). Bonferroni post-hoc comparisons were conducted when appropriate. Statistical significance was set at *p* ≤ 0.05.

## Results

### RmTBI induces persistent cognitive and motor deficits

**Figure 1:**
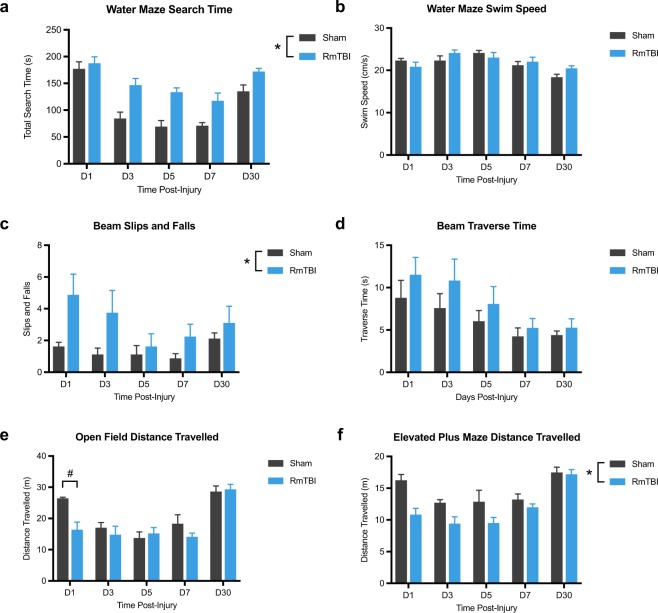
RmTBI induces persisting cognitive and sensorimotor deficits. In the water maze, the RmTBI rats had longer search times compared to sham rats (**a**), but did not differ from shams on the measure of swim speed (**b**). For the beam task, RmTBI rats had an increased number of slips and falls than sham rats (**c**), while all rats had faster traverse times as serial testing progressed (D). In the open field, RmTBI rats travelled less than sham controls at day 1 post-injury (**e**). In the elevated plus maze, the RmTBI rats travelled less compared to sham rats (**f**). *RmTBI rats significantly different than sham rats, p < 0.05. ^#^RmTBI rats significantly different than sham rats at day 1 recovery, p < 0.05. Mean ± SEM.

For the water maze, two-way ANOVA detected significant main effects of injury (F_1, 71_ = 40.51, *p* = 0.0001) and recovery time (F_4, 71_ = 23.30, *p* = 0.0001) on the measure of platform search time (Fig. 1a). Although all rats had improved performance as serial testing and recovery time progressed, the RmTBI rats had longer search times compared to sham controls. There were no significant differences between the RmTBI and sham rats for the measure of swim speed (*p* > 0.05; Fig. 1b), suggesting that motor deficits did not negatively affect the ability of the RmTBI rats to perform the task.

For the beam task, two-way ANOVA detected a significant main effect of injury (F_1, 71_ = 11.13, *p = *0.001; Fig. 1c), indicating that RmTBI rats had an increased number of slips and falls, regardless of recovery time. There was also a significant effect of recovery time (F_4, 71_ = 99.35, *p = *0.002; Fig. 1d) on the measure of traverse time, with all rats having faster traverse times with serial testing as recovery progressed.

In the open field, there was a significant injury x recovery time interaction (F_4, 71_ = 2.60, *p = *0.046; Fig. 1e), on the measure of distance travelled. Post-hoc analysis found that RmTBI rats travelled less than sham controls at day 1 post-injury. There were also significant main effects for injury (F_1, 71_ = 2.60, *p = *0.030; Fig. 1e) and recovery time (F_4, 71_ = 17.49, *p* = 0.0001; Fig. 1e).

For the elevated plus maze, two-way ANOVA detected significant main effects of injury (F_1, 71_ = 40.51, *p* = 0.0001; Fig. 1f) and recovery time (F_4, 71_ = 23.30, *p* = 0.0001; Fig. 1f) on the measure of distance travelled. Although all rats traveled less as serial testing and recovery time progressed, the RmTBI rats travelled less compared to sham rats. There were no differences between the RmTBI and sham rats on the measure of time spent in the open arm (*p* > 0.05; data not shown), suggesting that RmTBI did not affect anxiety-like behavior.

### RmTBI induces structural brain damage

**Figure 2:**
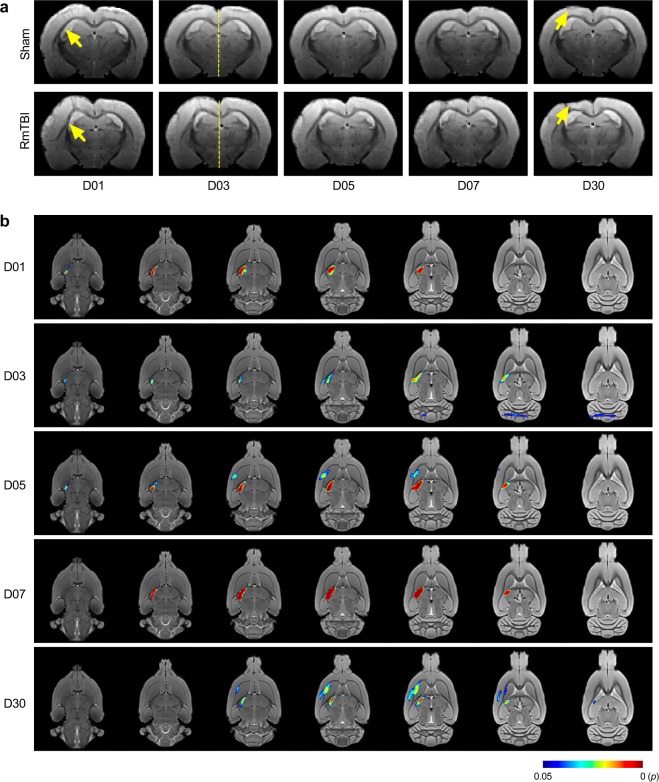
RmTBI induces structural brain damage. (**a**) T2*-weighted template images showing that RmTBI induces abnormalities in the ipsilateral hemisphere at each of the recovery times relative to sham controls. Yellow arrows point to regions of structural deformation and dashed lines emphasise mid-line shift. (**b**) TBM analysis at each time point indicates regions of significant deformation in the ipsilateral hemisphere in the RmTBI rats (FWE-corrected, p < 0.05).

As shown in the T2*-weighted template images in Fig. 2, RmTBI resulted in structural abnormalities in the ipsilateral hemisphere at each of the recovery times relative to sham controls (Fig. 2a). A tensor-based morphometry analysis at each time point supported this observation, revealing regions of significant deformation in the ipsilateral hemisphere in the RmTBI rats (FWE-corrected p < 0.05; Fig. 2b).

### RmTBI induces persistent changes on DWI biomarkers

**Figure 3:**
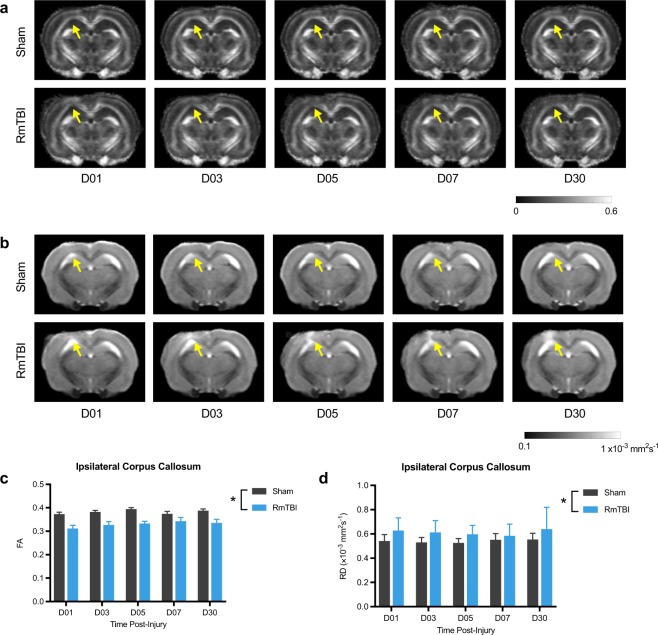
DTI abnormalities in the corpus callosum after RmTBI. Template FA (**a**) and RD (**b**) images for each of the recovery times. Regardless of recovery time, FA was reduced (**c**) and RD was increased (dD) in the ipsilateral corpus callosum of RmTBI rats compared to shams. *RmTBI rats significantly different than sham rats, p < 0.05. Mean ± SEM.

To assess RmTBI-induced injury to the corpus callosum, we conducted DTI and tractography analysis. For the DTI measure of FA, two-way ANOVA detected a significant effect of injury in the ipsilateral corpus callosum (F_1, 16_ = 18.67, *p* = 0.0005; Fig. 3c), indicating that FA was reduced in RmTBI rats compared to shams. For RD, two-way ANOVA detected a significant effect of injury in the ipsilateral corpus callosum indicating that RD was increased in RmTBI rats compared to shams (F_1, 16_ = 6.15, *p* = 0.025; Fig. 3d).

**Figure 4:**
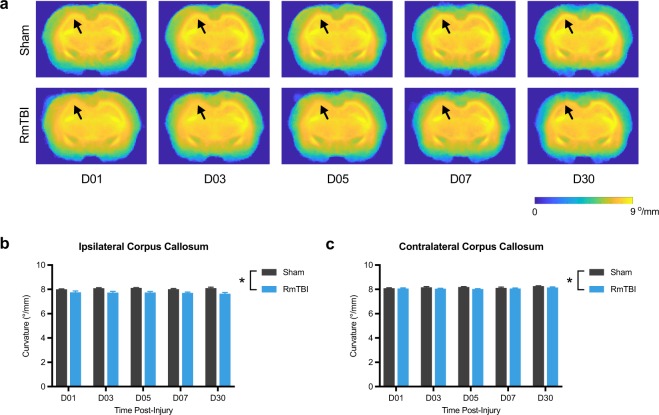
Mean curvature in the corpus callosum is reduced after RmTBI. (**a**) Template mean curvature images for each of the recovery times. Regardless of recovery time, RmTBI rats had reduced mean curvature in both the ipsilateral (**b**) and contralateral (**c**) corpus callosum compared to sham controls. *RmTBI rats significantly different than sham rats, p < 0.05. Mean ± SEM.

For the tractography measures, two-way ANOVA detected a significant effect of injury in the ipsilateral corpus callosum (F_1, 16_ = 2.75, *p* = 0.002; Fig. 4b) and contralateral corpus callosum (F_1, 16_ = 5.71, *p* = 0.03; Fig. 4c) on the measure of mean curvature; with RmTBI rats having reduced mean curvature in both.

**Figure 5:**
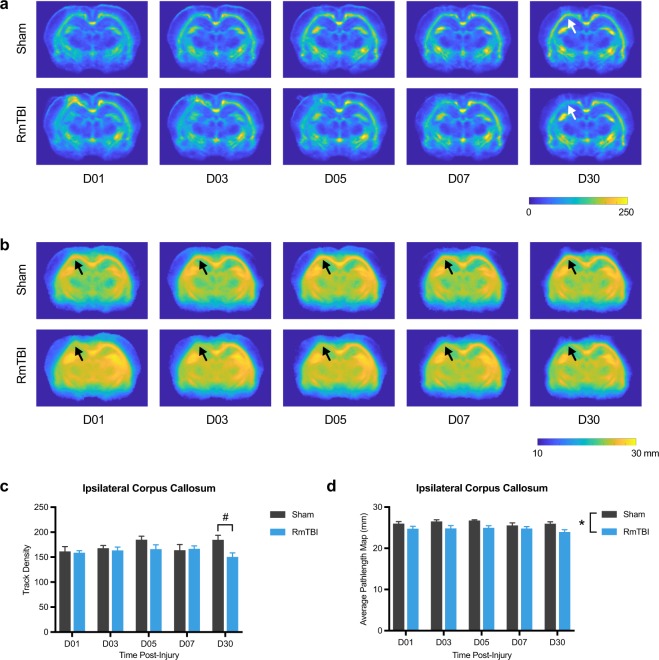
TDI and APM in the corpus callosum are reduced after RmTBI. Template TDI (**a**) and APM (**b**) images for each of the recovery times. (**c**) RmTBI rats had reduced TDI at day 30 recovery compared to sham controls. (**d**) Regardless of recovery time, RmTBI rats had reduced APM in the ipsilateral corpus callosum compared to sham controls. *RmTBI rats significantly different than sham rats, p < 0.05. ^#^RmTBI rats significantly different than sham rats at day 30 recovery, p < 0.05. Mean ± SEM.

For TDI, two-way ANOVA detected a significant injury x time interaction in the ipsilateral corpus callosum (F_4, 64_ = 2.63, *p* = 0.042; Fig. 5c), and post-hoc analysis revealed that TDI was significantly reduced in the ipsilateral corpus callosum of RmTBI rats compared to shams at D30 (*p* = 0.01).

For APM, two-way ANOVA detected a significant effect of injury in the ipsilateral corpus callosum (F_1, 16_ = 5.89, *p* = 0.03; Fig. 5d) with RmTBI rats having reduced APM regardless of time.

### RmTBI alters the levels of protein biomarkers in the plasma

**Figure 6:**
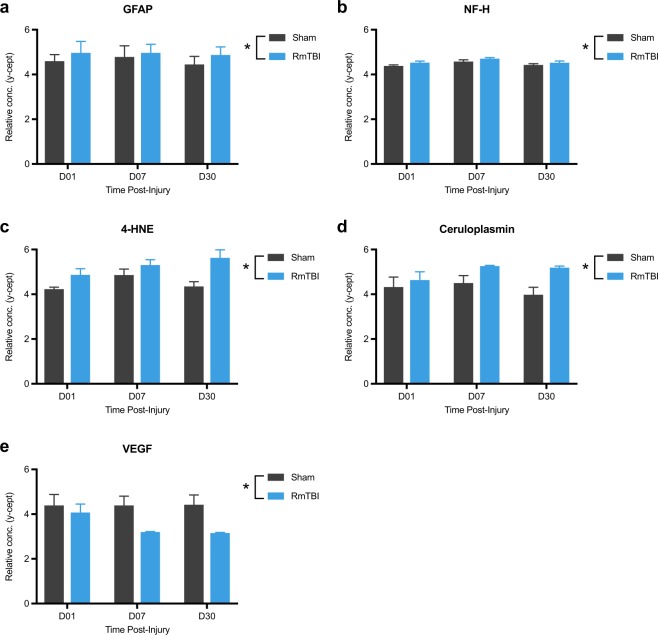
RmTBI alters the levels of protein biomarkers in the plasma. Regardless of recovery time, RmTBI rats had significantly elevated plasma levels of GFAP (**a**), NF-H (**b**), 4-HNE (**c**), and ceruloplasmin **(d**), while levels of VEGF were decreased (**e**) as compared to shams. *RmTBI rats significantly different than sham rats, p < 0.05. Mean ± SEM.

Two-way ANOVA revealed that RmTBI rats had significantly elevated plasma levels of GFAP (F_1, 30_ = 5.27, *p* = 0.029; Fig. 6a), NF-H (F_2, 38_ = 9.66, *p* = 0.004; Fig. 6b), 4-HNE (F_1, 24_ = 10.49, *p* = 0.003; Fig. 6c), and ceruloplasmin (F_1, 29_ = 8.93, *p* = 0.006; Fig. 6d), while levels of VEGF were decreased (F_1, 37_ = 10.71, *p* = 0.002; Fig. 6e) as compared to shams. No statistically significant differences in plasma levels of S100β, NSE or Tau were observed (data not shown).

## Discussion

The aim of this study was to assess the utility of MRI and select plasma protein biomarkers, as well as neurobehavior measures, to detect acute, sub-acute, and long-term changes in rats that received two mTBIs. Consistent with previous studies that have administered RmFPI separated by a five-day inter-injury time^26–28^, RmFPI rats in this study were found to have lasting sensorimotor and cognitive impairments, as demonstrated by increased slips/falls on the beam task and longer search latencies to locate the hidden platform during the water maze. Also consistent with previous studies, RmTBI rats had evidence of long-term brain damage in grey and white matter structures^10,26,28,44^. T2*-weighted template images showed evidence of a midline shift, from the ipsilateral to contralateral hemisphere, as well as deformation of the ipsilateral cortex, hippocampus, and corpus callosum. There was also clear loss of cortical tissue that was evident at D30 post-injury. These results were supported by a TBM analysis that revealed significant, and lasting changes in the ipsilateral hemisphere. Moreover, DWI analysis found that RmTBI rats had abnormalities on a number of DTI (reduced FA and increased RD) and tractography (reduced mean curvature, TDI, and APM) metrics in the corpus callosum suggestive of axonal injury^12,14–16^. The brain damage detected by MRI may help account for the behavioral deficits seen in the RmTBI rats. For example, the RmFPI impact site is adjacent to the sensorimotor cortex, and the observed cortical atrophy may explain the persistent sensorimotor deficits on the beam task^26,28,45^. Furthermore, the structural changes in the hippocampus, which plays significant roles in memory, cognition, and spatial navigation, may contribute to the observed deficits in the water maze task^46^.

RPPM analysis of plasma protein biomarkers found changes in RmTBI rats indicative of axonal injury (increased NF-H)^18–20,40^, astroglia damage (increased GFAP)^18–20,47^, vascular irregularities (decreased VEGF)^18–20,38^, oxidative stress (increased 4-HNE)^39^, and metabolic dysfunction (increased ceruloplasmin)^18–20,36^. These findings support the use of plasma protein biomarkers to identify changes following RmTBI, and to provide insight into the mechanisms that underlie the brain damage observed in these rats. Elevated levels of circulating NF-H is considered a marker of axonal injury^40,48^. The current findings that NF-H levels remain elevated until at least D30 post-RmTBI are consistent with the changes in DWI measures, and together indicate that RmFPI induces lasting and perhaps progressive axonal injury. VEGF stimulates angiogenesis and neurogenesis^38,49^. Previous studies have found that inhibition of VEGF signaling results in increased cell death after TBI^50^, while treatment with VEGF is neuroprotective^51^. Therefore, the present findings of a lasting decrease in VEGF in the presence of behavioral impairments and brain injury are consistent with the notion that VEGF may play a beneficial role in recovery after neurotrauma^38^. Ceruloplasmin is involved in the transport of free iron across the cell membrane and in iron metabolism^36,52,53^. Increased levels found in the RmTBI rats may be a response to increased iron levels that can be induced by TBI due to bleeding (i.e., microvascular damage) and/or disruptions in ion homeostasis^54–56^. Excess iron levels can also trigger neuroinflammation and the production of reactive oxygen species^57,58^, both of which are mechanisms implicated in neurodegeneration and may help explain the observed increases in 4-HNE^56,59^.

As alluded to in the introduction, we previously investigated behavior, MRI, and plasma protein outcomes after a single mFPI at the same recovery times studied in the present study [12]. For the behavior measures, the only statistically significant behavioral difference found in the previous study was that a single mFPI resulted in water maze deficits at day 3 post-injury, and these resolved to sham-injured levels by day 5. This is in stark contrast to the persisting water maze and beam deficits observed in the RmTBI rats in the current study. For the MRI outcomes, a single mFPI did not result in significant volumetric differences in the cortex, hippocampus, corpus callosum, or lateral ventricles compared to sham-injured, but did induce changes on DWI measures at days 1, 3, 5 and 30 post-injury. In comparison, the present study found evidence of structural MRI changes after RmTBI, with a midline shift as well as deformation of the ipsilateral cortex, hippocampus, and corpus callosum. On the DWI measures, there was evidence of differences between the RmTBI and sham groups at each of the recovery times, and there appears to be more robust differences on DWI measures at day 30 post-injury in the RmTBI rats relative to the single mFPI. However, it is important to consider that the analyses of the MRI measures differed between the studies, with the present paper using updated methodologies. For the plasma protein markers, a single mFPI was previously found to induce increased levels of ceruloplasmin and NF-H at day one post-injury, decreased tau at day seven post-injury, and decreased VEGF at days seven and thirty post-injury. Here, we also found changes on the same markers, though these changes were present across each of the recovery times. Additionally, the current study found a statistically significant increase of 4-HNE in the RmTBI rats compared to sham-injury, which was not found in the single mFPI study.

Findings from the current RmTBI study, combined with findings from our previous single mFPI study^12^, may also provide insight into mechanisms that underlie increased cerebral vulnerability (ICV) to RmTBI. Growing evidence suggests that the long-term deleterious effects of RmTBI may be due to sustaining a RmTBI before the brain has recovered from the initial mTBI and is in a period of ICV^60,61^. We, as well as others, have previously found that a single mFPI results in transient behavioral abnormalities, with no evidence of major structural brain damage or significant neuronal loss^2,10,12,62^. Of particular relevance, we previously found that the behavioral deficits induced by a single mFPI recover to sham levels by day five post-injury (i.e., the time when a second mFPI was given in the current study), and that conventional structural MRI was unable to detect differences between the single mFPI and sham groups at that time^12^. However, the single mFPI rats showed changes on advanced DWI (e.g., axonal injury) and plasma levels of protein biomarkers (e.g., VEGF) that persisted beyond the resolution of behavioral changes^12^. Accordingly, our current findings of lasting neurological abnormalities after a second mFPI, given in the presence of DWI and plasma biomarker changes induced by the first mTBI, may provide insight into pathophysiological changes that underlie ICV^60^. For example, angiogenesis post-TBI may help restore the supply of blood and oxygen to damaged tissue^63^. If this process has been compromised in mFPI rats, as decreased VEGF suggests, the brain may be more vulnerable if exposed to a subsequent insult.

The current findings may also provide insight into potential therapeutic avenues. The VEGF and ceruloplasmin findings suggest neurovascular injury, therefore interventions that upregulate angiogenesis or VEGF may mitigate ICV and the consequences of RmTBI. Notably, there is recent evidence that exercise, which is known to increase VEGF and angiogenesis amongst other things^64^, is beneficial after mTBI^65,66^ and it would be of interest to investigate the precise biological mechanisms that drive this effect. The DWI and NF-H biomarker findings suggest axonal injury as a central pathophysiology in RmTBI, and a number of biochemical mechanisms may contribute to this. For example, oxidative stress and neuroinflammation can contribute to axonal injury, and findings from the current (i.e., elevated 4-HNE and GFAP) and previous studies suggest that oxidative stress and neuroinflammation are present after mTBI^24,26–28^. As such, interventions targeting these mechanisms may mitigate ICV and axonal injury in RmTBI. With that said, it is important to note that this discussion on underlying mechanisms/ICV and therapies is largely speculative, as a limitation of the current study is the lack of outcomes that directly assessed neuropathology. Although the biomarker methods used in this study are translatable to the clinical setting, ideally the changes in MRI and plasma protein measures would be compared against direct measures of neuropathophysiology. For example, histological studies that examined axonal integrity (e.g., myelin staining) and neurovascular injury would greatly complement the current biomarker findings. Taken together, future studies are still required to determine the precise neuropathological mechanisms that underlie the biomarker findings and ICV; to ascertain whether the biomarkers can be used to determine safe return to play/duty decisions and vulnerability to develop neurodegenerative changes; and to investigate intervention options to improve recovery.

A limitation of this study that should be acknowledged is that the mFPI model of mTBI is not optimal as it requires the use of anesthetics and surgery (i.e., a craniotomy), both of which have the potential to confound behavior, MRI, and plasma protein results^21,67–69^. Furthermore, the physical properties of the rodent brain relative to the human brain likely influences the nature of the brain injury. For example, the rat brain has a smooth surface as compared to the human brain with its many sulci and gyri. As such, the smooth brain of the rat may serve as a plate of compression on underlying structures whereas the gyri and sulci in humans may disperse the concussive forces in a different manner. These factors may account for the exacerbated acute and sub-acute brain injury observed in the RmTBI rats in this study relative to what would be expected in humans who have sustained RmTBI. Although the use of the mFPI model is justifiable by previous studies showing that single and repeated mFPI can result in behavioral and pathophysiological changes^24,25,62,70^, in a manner that is largely consistent with what occurs in the clinical setting^2^, newer models of mTBI/concussion that avoid invasive procedures and anesthetics are now being developed^10,71–73^. For example, our group and others have recently developed an awake closed head injury model of concussion that avoids many of the abovementioned confounding variables^71–73^. Therefore, while the current findings provide important insights into potential biomarkers for RmTBI, future studies should strive to replicate the current findings in next generation mTBI models, as well as in human participants^21^.

In conclusion, how RmTBI affects MRI and plasma protein biomarkers, and how these relate to the functional consequences of RmTBI, is not well understood. To that end, this study has characterized the changes in brain microstructure detected by MRI, plasma levels of protein biomarkers using proteomics, and neurobehavioral outcomes at different recovery times after RmTBI in rats. We found that rats given RmTBI had persistent cognitive and sensorimotor deficits, abnormalities on structural MRI and DWI measures, as well as increased plasma levels of protein markers indicative of axonal injury, glial reactivity, oxidative stress, vascular irregularities, and mitochondrial and metabolic dysfunction. Although future studies are required, these findings provide additional evidence that RmTBI can result in long-term neurological deficits, offer insight into the pathophysiological mechanisms that contribute to these consequences, and support the use of MRI and plasma proteomics to objectively detect abnormalities after RmTBI.
